# HIF-1α, HIF-2α, and ProExC: diagnostic or prognostic relevance in conjunctival intraepithelial neoplasia?

**DOI:** 10.1007/s00417-020-04734-4

**Published:** 2020-05-26

**Authors:** Simone Nuessle, Daniel Soriano, Daniel Boehringer, Hans Mittelviefhaus, Clemens Lange, Thomas Reinhard, Lisa Atzrodt, Claudia Auw-Haedrich

**Affiliations:** Eye Center, Medical Center, Faculty of Medicine, University of Freiburg, Killianstr. 5, 79106 Freiburg im Breisgau, Germany

**Keywords:** Conjunctival intraepithelial neoplasia, HIF, ProExC, immunohistochemistry

## Abstract

**Purpose:**

The aim of this study was to investigate HIF-1α, HIF-2α, and ProExC expression in conjunctival intraepithelial neoplasia (CIN), to differentiate between metaplasia and dysplasia, and to access their value as diagnostic and prognostic immunohistochemical markers. Recurrence and progression into SCC (squamous cell carcinoma) were defined as endpoints.

**Methods:**

Forty-three specimens including CIN I (2), CIN II (9), CIN III (29), with and without metaplasia, and metaplasia alone (3), as well as 21 conjunctival control specimens, were stained with antibodies against HIF-1α, HIF-2α, and ProExC. The percentage of positively stained cells were calculated and used for further analysis.

**Results:**

The mean percentages of HIF-1α and HIF-2α were not increased in CIN. In comparison, the expressions of these markers were even significantly elevated in control specimens (*p* < 0.001). Upper epithelial cells in CIN were more often ProExC-positive compared with normal conjunctiva or metaplasia (*p* = 0.06 and *p* = 0.07). Cox proportional-hazards analysis was performed for characterization of factors influencing the combined endpoint and showed a significant elevated hazard ratio for staining with ProExC (*p* = 0.04) compared with HIF-1α (*p* = 0.26) and HIF-2α (*p* = 0.49).

**Conclusion:**

Our study shows that HIF-1α and HIF-2α do not serve as diagnostic or prognostic markers in CIN. ProExC seems to be a potential indicator for CIN, but not a reliable diagnostic marker. However, control specimens occasionally also display a high percentage of ProExC-positive cells and staining over the entire epithelial layer.

## Introduction

Conjunctival intraepithelial neoplasia (CIN) and squamous cell carcinoma (SCC) are rare tumors but are among the most common conjunctival malignancies with an incidence of SCC between 0.03/100000 persons per year in the USA and 3.4 and 3.0 cases/year/100000 in Zimbabwe [1, 2].

CIN can present as a simple dysplasia to carcinoma in situ. The diagnosis of dysplasia and the grading of CIN are made histopathologically by means of subjective assessment. Classification and grading parallel cervical intraepithelial neoplasia (cervical IN). CIN grade depends on the proportional distribution of dysplasia in relation to the epithelial height [3]. By analogy to the cervical IN, there are three different grades which range from CIN grade I with dysplasia of the basal third to CIN grade III or carcinoma in situ with dysplasia over 2/3 of the whole thickness of the epithelium [4].

Dysplasia is defined by morphological criteria such as atypical, enlarged, and/or hyperchromatic nuclei; increased mitosis; and apoptosis. These criteria are in some extent quite subjective. Therefore, additional immunohistochemical stains supporting malignancy would be helpful, also in differentiating those tumors consisting of metaplasia alone from those with CIN and additional metaplasia.

In cervical IN, the markers HIF-1α and ProExC have already been evaluated as diagnostic markers [5–7].

**Fig. 1:**
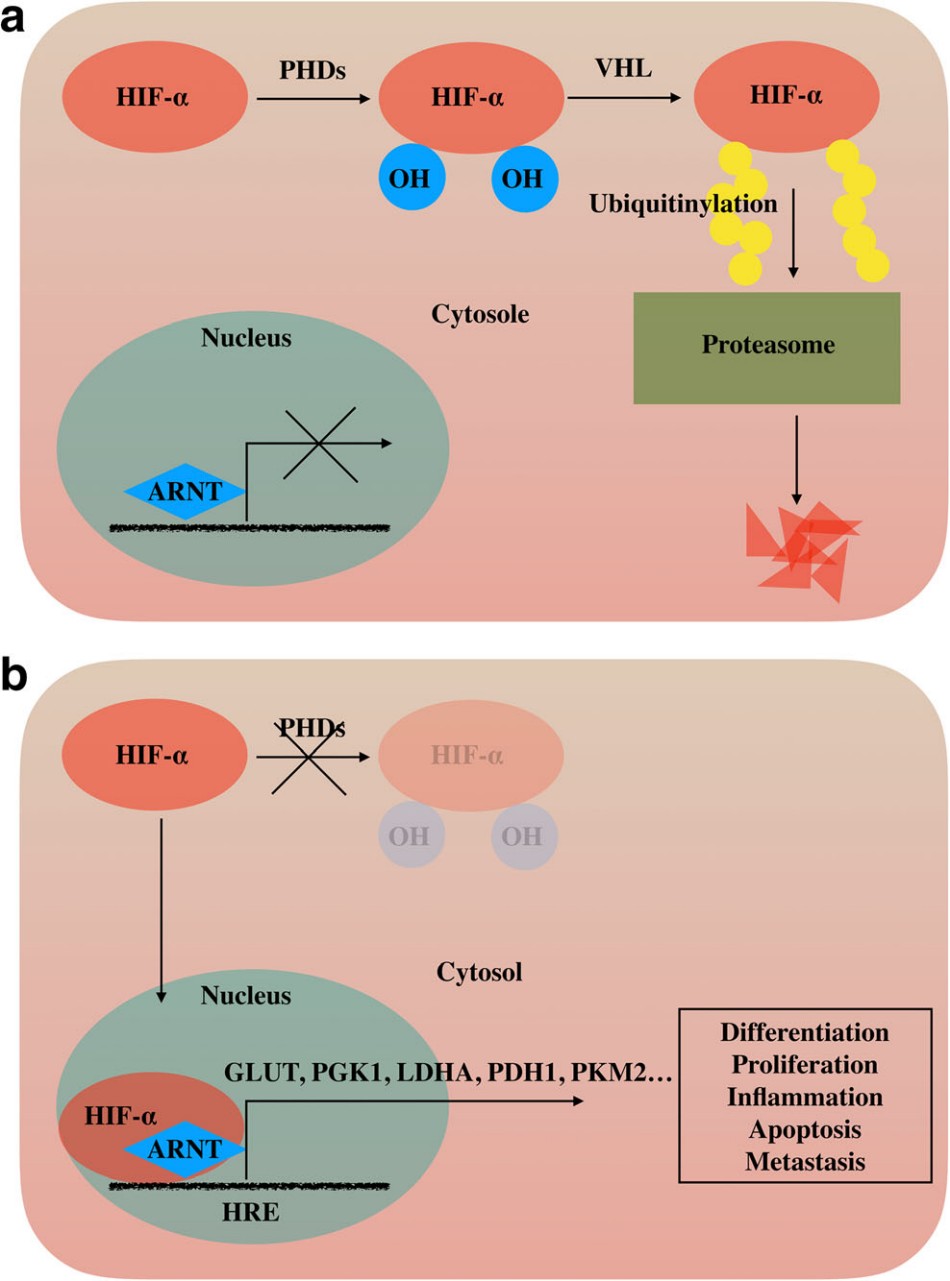
Schematic representation of the oxygen-dependent degradation of HIF-α by ubiquitination (modified from Rankin and Giaccia [9])

A higher expression of HIF (hypoxia-inducible factors) was linked to poorer prognosis in a large number of different tumors [7–9]. HIFs, mainly HIF-1α and HIF-2α, are heterodimeric transcription factors which are rapidly degraded under normoxic conditions by hydroxylation (PHD 1–3), binding to the VHL (von Hippel Lindau) protein and together marked by E3-ubiquitin-ligase complex before proteasomal degradation (Fig. 1). HIF is known to have a key role in cellular responses to hypoxia and is an important regulator of tumorigenesis including proliferation, differentiation, angiogenesis, immortalization, metastasis, and apoptosis [10–13].

In cervical IN, studies observed a higher expression of HIF-1α in high-grade cervical IN compared with that in normal cervical epithelium [5, 7]. Furthermore, No et al. also showed higher expression of HIF-1α in high-grade in comparison with low-grade dysplasia.

ProExC consists of two monoclonal antibodies against TOP2A (topoisomerase 2α) and MCM2 (minichromosome maintenance protein 2) which are two important factors in DNA replication [14, 15]. ProExC was shown to indicate a correlation between expression and cervical IN grades with significant higher expression of higher cervical IN grades whereas specimens with metaplasia showed less ProExC expression [6].

Due to the similarity between cervical and conjunctival intraepithelial neoplasia, we focused on the markers HIF-1α and ProExC as potential diagnostic and prognostic markers of CIN. Besides this, we aimed to investigate HIF-2α in CIN which level of expression has been found increased in various tumors like bladder, breast, colon, and also cervical carcinomas [9].

## Material and methods

The study was approved by the ethics committee of the Albert-Ludwig University Freiburg, Germany; written informed consent was obtained from the study participants. Forty-three conjunctival specimens with diagnosed CIN or metaplasia (details in results) which had been excised at the Eye Center Freiburg between 2004 and 2013 were included. Control specimens originated from excess conjunctiva that had been resected at the end of buckle retinal detachment surgeries.

### Preparation of the specimen

After excision, the specimens were fixed in 4% formaldehyde in phosphate buffer of 7.0 pH for 24–48 h, dehydrated in increasing concentrations of ethanol (70–100%) and xylol and infiltrated with paraffin (TP 1020, Leica, Wetzlar, Germany). Sections of 4 μm thickness were cut and floated on deionized water at 20 °C, and single sections were mounted on X-tra adhesive glass slides (Leica, Wetzlar, Germany). Slides were stretched in a water bath at 47–48 °C, subsequently dried at 60 °C for 30 min, and stored at a room temperature in darkness.

Prior to immunohistochemical staining, the specimens were deparaffinized and rehydrated by xylol and in decreasing concentrations of ethanol (100–70%). Afterward, they were demasked using a citrate buffer of pH 6, heated in deionized water at 95 °C for 20 min, and slowly cooled down for 20 min. Now, they were stained immunohistochemically using the catalyzed reporter deposition method by biotin-free tyramide amplification with the DAKO CSA II kit and washed with 0.05 M TBST-wash buffer between the steps as recently described [16]. First, the endogen peroxidase was blocked by 3% hydrogen peroxide for 15 min and the slides were incubated in 0.015 M sodium azide buffer before 100 μl of the diluted mouse monoclonal antibodies (pre-diluted ProExC, HIF-1α 1:8000 and HIF-2α 1:500) were applied for 15 min at a room temperature and 18–21 h at 4 °C. Applied were the secondary antibody from the DAKO CSA II kit for 60 min, the fluorescein-tyramide and hydrogen peroxide for amplification for 15 min in the dark, the tertiary antibody from the DAKO CSA II kit for 15 min, and chromogen AEC under microscopical control of the positive control 4 min for ProExC and 10 min for HIF before counterstaining with Harris hematoxylin for 2 s.

### Evaluation of the specimens

Two to five representative sections of each specimen were photographed at a magnification of × 400 (HIF) and × 200 (ProExC), respectively. The cell number was determined by a self-programmed, validated, automated, cell counting software (reliability of 100%). Positive nuclear staining for HIF-1α and HIF-2α, and a strong cytoplasmic staining for HIF-2α were determined by counting manually, whereas positively stained cells for ProExC were counted automatically by the computer-based calculator. The percentage of positively stained cells for HIF-1α, HIF-2α, and ProExC was calculated. The results were subdivided into three groups: strongly (> 1.10% (HIF-1α), > 9.38 % (HIF-2α), and > 59.19% (ProExC) positively stained cells), medium (0.43–1.10% (HIF-1α), 1.27–9.38% (HIF-2α), and 43.5–59.19% (ProExC) positively stained cells), and weakly (< 0.43% (HIF-1α), < 1.27% (HIF-2α), and < 43.5% (ProExC) positively stained cells) expressed. In addition, the distribution of positively stained cells for ProExC regarding the vertical extension of epithelial thickness was determined and divided into basal, middle, and upper third.

To investigate whether inflammation might have an impact on the immunoreaction, we also counted manually all intraepithelial as well as subepithelial inflammatory cells (lymphocytes) in each photograph. Four groups were defined as 0: no inflammatory cell, 1: < 5 inflammatory cells, 2: 5–20 inflammatory cells, and 3: > 20 inflammatory cells.

We also reviewed the medical records for the following: age at time of surgery, sex, histopathological diagnosis, and days until tumor-associated events (recurrence and SCC development, which were combined for further analysis).

We fitted a proportional hazards Cox regression model in order to assess the influence of the aforementioned covariates on tumor-related events. Significant level was set at *p* value < 0.05.

## Results

### Baseline data

**Fig. 2:**
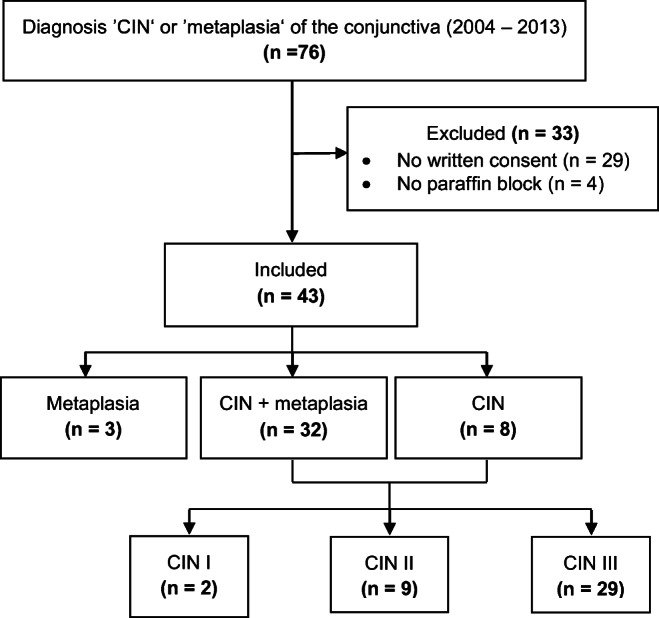
Flow chart of baseline data

The study cohort comprised 12 women and 31 men with a median age of 63 years (27–87 years) at time of surgery. Of these 43 included specimens, 3 were diagnosed with metaplasia alone, 8 with CIN without metaplasia, and 32 with CIN and metaplasia. Referring to the CIN specimen, 2 were classified as CIN I, 9 as CIN II, and 29 as CIN III. The median follow-up period was 1013 days (interquartile range, 486–2075 days). None of the included patients developed metastases or died in association with the included disease. Recurrence was shown in 9 patients (23%) after a median time of 301 days (range, 110–2075 days), most of them within the first 2 years. One was initially diagnosed with CIN I, 2 with CIN II, and 6 with CIN III. Three patients (7%) developed SCC after 441, 806, and 2653 days. All of them were male patients with initial diagnosis of CIN III and concomitant autoimmune dermatitis. In metaplasia, there was—as expected—no tumor-associated event. The control group comprised 21 normal conjunctival specimens from 9 female and 12 male patients with a median age of 59.5 years (33–82 years) (Fig. 2 and Table 1).

**Table 1:** Baseline data of the 43 cases included in the study

No.	Age (years)	Sex	Histological diagnosis	Follow-up time (days)	Recurrence of CIN	Progression to SCC
1	66.6	f	Metaplasia	3808	No	No
2	52.7	m	Metaplasia	3332	No	No
3	63.0	m	Metaplasia	2692	No	No
4	52.0	m	CIN III + metaplasia	2653	Yes	Yes
5	54.1	m	CIN III	441	Yes	Yes
6	51.2	m	CIN III + metaplasia	806	No	Yes
7	70.2	m	CIN III + metaplasia	1789	Yes	No
8	63.40	m	CIN II	859	Yes	No
9	49.1	m	CIN III + metaplasia	255	Yes	No
10	37.0	f	CIN I + metaplasia	209	Yes	No
11	48.2	m	CIN III + metaplasia	2075	Yes	No
12	46.4	m	CIN II + metaplasia	279	Yes	No
13	71.3	m	CIN III	789	Yes	No
14	65.0	m	CIN III + metaplasia	4465	No	No
15	76.6	f	CIN III + metaplasia	1526	No	No
16	62.6	m	CIN II + metaplasia	2555	No	No
17	70.8	m	CIN III + metaplasia	967	No	No
18	46.5	f	CIN III + metaplasia	1383	No	No
19	69.4	m	CIN III + metaplasia	0	No	No
20	87.1	m	CIN III + metaplasia	3109	No	No
21	66.4	f	CIN I + metaplasia	3040	No	No
22	54.5	m	CIN III + metaplasia	1561	No	No
23	70.2	m	CIN III + metaplasia	258	No	No
24	78.4	m	CIN III	774	No	No
25	55.2	m	CIN III + metaplasia	803	No	No
26	67.4	f	CIN II	2438	No	No
27	42.9	m	CIN III	2617	No	No
28	27.9	m	CIN II + metaplasia	851	No	No
29	77.6	f	CIN III + metaplasia	1883	No	No
30	82.4	m	CIN III + metaplasia	218	No	No
31	38.9	f	CIN III + metaplasia	1342	No	No
32	29.2	f	CIN III	1502	No	No
33	72.5	m	CIN III + metaplasia	1138	No	No
34	63.0	f	CIN III + metaplasia	798	No	No
35	67.4	m	CIN II + metaplasia	486	No	No
36	73.7	m	CIN II + metaplasia	1557	No	No
37	62.4	f	CIN III + metaplasia	978	No	No
38	68.0	m	CIN III + metaplasia	2	No	No
39	62.3	m	CIN II	1013	No	No
40	43.3	m	CIN III + metaplasia	293	No	No
41	82.1	m	CIN III + metaplasia	1258	No	No
42	65.1	m	CIN III + metaplasia	1	No	No
43	63.4	f	CIN II + metaplasia	811	No	No

### Percentage HIF-1α, HIF-2α, and ProExC expression

**Fig. 3:**
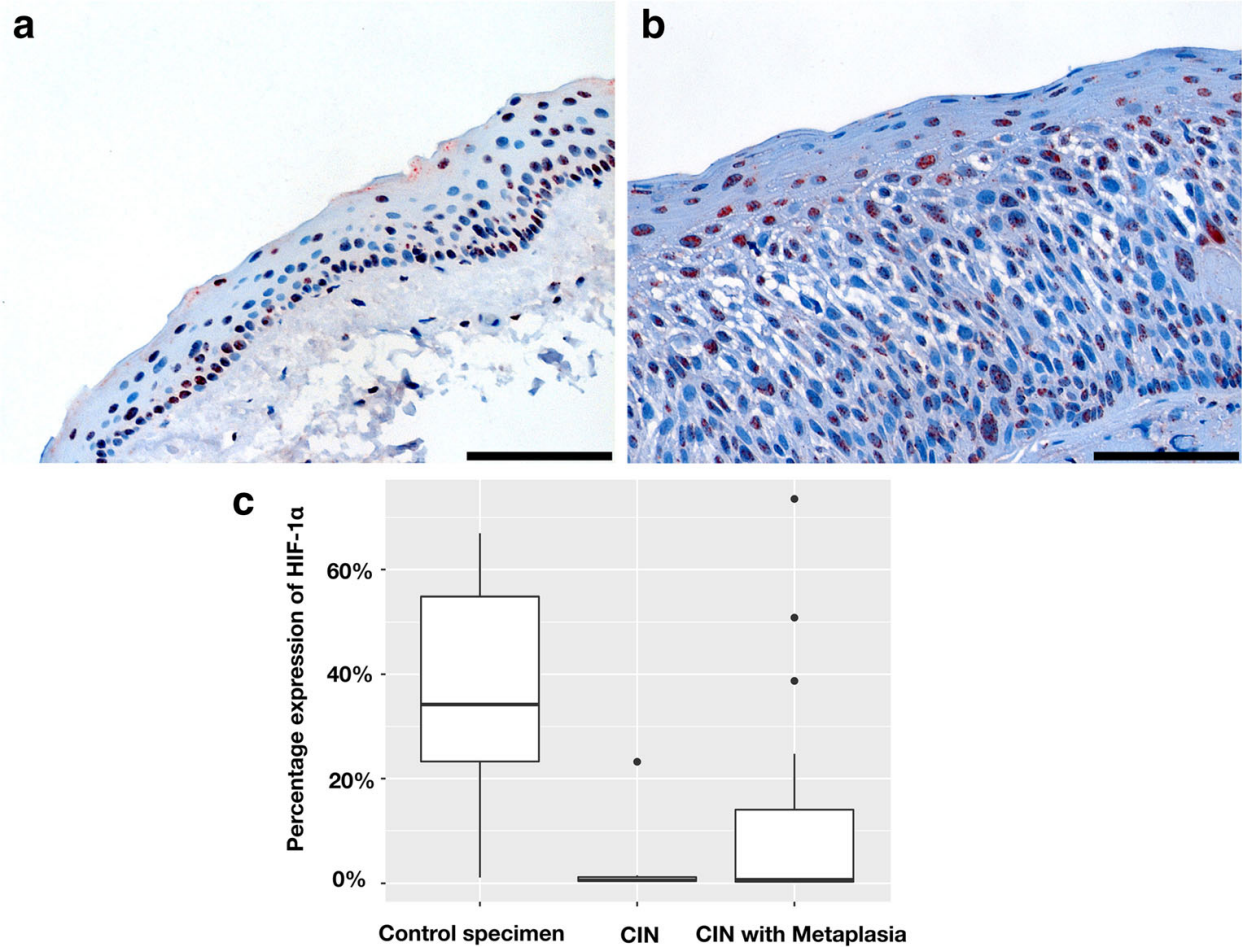
HIF-1α: **a** + **b** Representative immunohistochemical nuclear staining (red) for HIF-1α (scale bar = 100 μm). **a** Nuclear staining in normal conjunctiva and **b** in CIN III with metaplasia. **c** Boxplot for percentage expression of HIF-1α compared with controls, CIN, and CIN with metaplasia (*p* < 0.001)

The mean percentages of HIF-1α-positive cells in all CIN specimens with or without metaplasia were 10.9% ± 3.0% and 3.9% ± 8.5%, respectively. Notably, HIF-1α expression was significantly elevated in normal conjunctival specimens compared with CIN without or with metaplasia with a mean percentage of 59.5% ± 20.0% HIF-1α-positive cells (*p* < 0.001) (Fig. 3c).

**Fig. 4:**
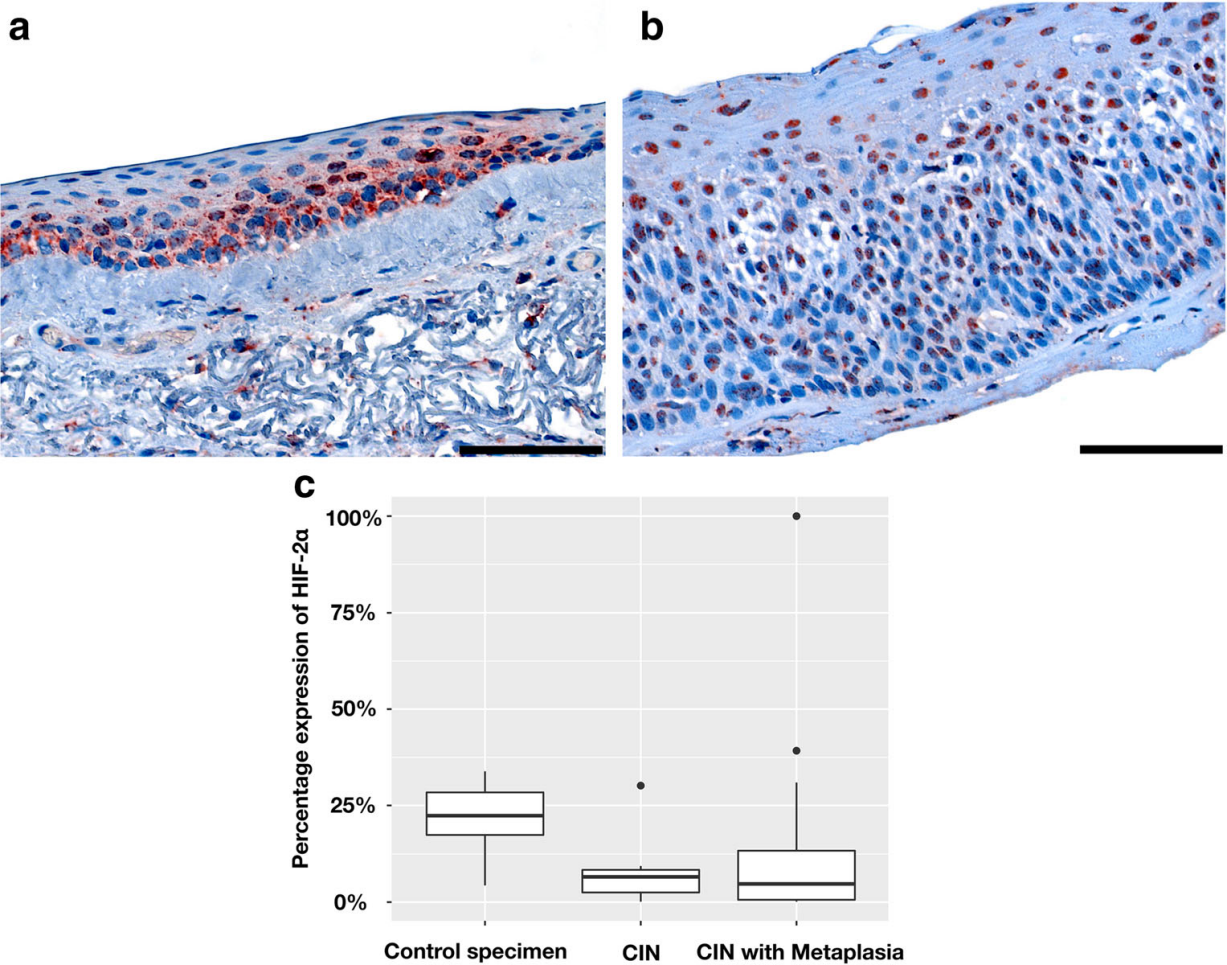
HIF-2α: **a** + **b** Representative immunohistochemical nuclear and cytoplasmic staining (red) for HIF-2α (scale bar = 100 μm). **a** Nuclear staining in normal conjunctiva and **b** in CIN III with metaplasia. **c** Boxplot for percentage expression of HIF-1α compared with controls, CIN, and CIN with metaplasia (*p* < 0.001)

The mean proportion of HIF-2α expression in all CIN specimens with or without metaplasia was calculated at 11.0% ± 19.5% and 8.4% ± 10.2%, respectively. As already shown in HIF-1α expression, the HIF-2α expression as well was significantly elevated in the control group compared with CIN specimen with a mean percentage of 20.1% ± 7.5% (*p* < 0.001) (Fig. 4c).

The proportion of ProExC-positive cells in CIN was similar to the control group with a mean percentage of 50.6% ± 23.1% in CIN without metaplasia, 50.2% ± 20.9% in CIN with metaplasia, and 45.2% ± 17.3% in control specimens (*p* = 0.55).

Our analysis showed that there was no correlation between extent of inflammation and immunostaining (*R*^2^ < 0.04).

### Vertical extension of ProExC

With regard to vertical extension of ProExC, we observed a correlation between the classification and the positively stained epithelial height, however missing statistical significance. About 52% of the normal conjunctival specimens showed positive staining for ProExC particularly in the basal third of the epithelium. In about 60% of the specimens with CIN, in contrast, cells in the upper third of the epithelium expressed ProExC (*p* = 0.06).

**Fig. 5:**
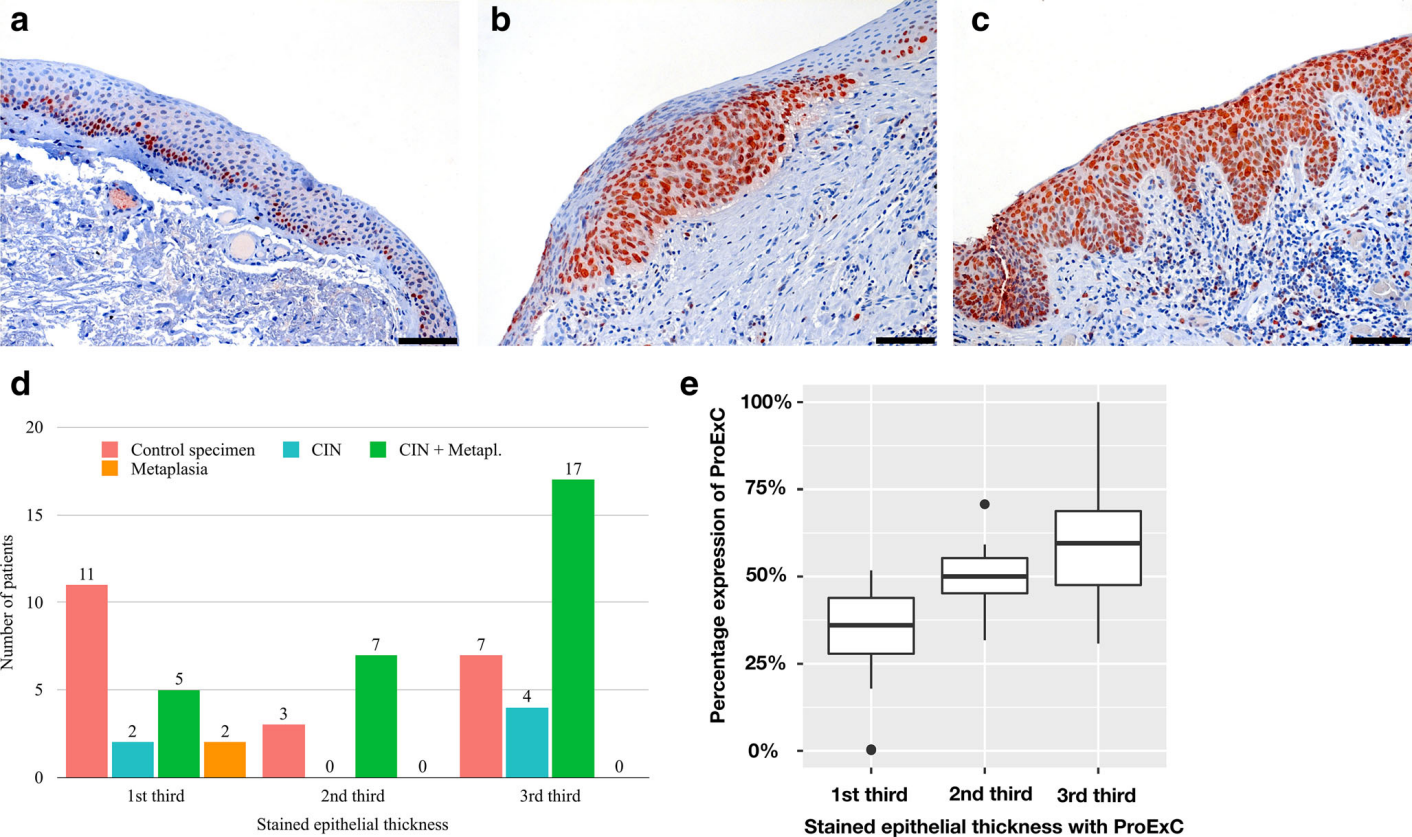
ProExC: **a**–**c** Representative immunohistochemical nuclear staining (red) for ProExC in different vertical extension (magnification × 200, scale bar = 100 μm). **a** Nuclear staining in the basal third of the epithelium in normal conjunctiva, **b** in the basal 2/3 of the epithelium in CIN grade II, and **c** of the whole thickness of the epithelium in carcinoma in situ. **d** Level of maximal (basal/middle/upper third = 1st/2nd/3rd) stained epithelial thickness with ProExC. Comparison between the classifications. **e** Correlation between the percentage expression of ProExC and the stained epithelial thickness (*p* < 0.001)

Moreover, we observed a statistically significant correlation between the percentage expression of ProExC and the level of stained epithelial thickness (*p* < 0.001) (Fig. 5e).

### HIF and ProExC expression and tumor-related events

No statistically significant difference of HIF-1α and HIF-2α expression was found between CIN specimen developing recurrence or progressing to SCC compared with CIN without any of these tumor-related events. Notably, the mean expression of HIF-1α in specimens with CIN recurrence and progression to SCC were even lower (Table 2).

**Table 2:** Proportion of HIF-1α-, HIF-2α-, and ProExC-positive cells

		*n*	Mean	*p* value
HIF-1α	CIN with recurrence	8	3% ± 6%	0.75
	CIN without recurrence	30	10% ± 18%	
	CIN with progression to SCC	3	1% ± 1%	0.58
	CIN without progression to SCC	35	10% ± 17%	
HIF-2α	CIN with recurrence	7	12% ± 16%	0.59
	CIN without recurrence	30	10% ± 19%	
	CIN with progression to SCC	3	4% ± 3%	0.55
	CIN without progression to SCC	34	11% ± 19%	
ProExC	CIN with recurrence	8	60% ± 22%	0.12
	CIN without recurrence	29	48% ± 20%	
	CIN with progression to SCC	3	70% ± 29%	0.19
	CIN without progression to SCC	34	49% ± 20%	

In the Cox regression, we observed a higher ProExC expression in specimens with CIN recurrence (*p* = 0.12) and progression to SCC (*p* = 0.19) compared with those without any tumor-related event, but without statistical significance. Moreover, all three specimens with progression to SCC during the observation period were stained up to the upper third of the epithelium. Nevertheless, the difference of those cases without progression to SCC was not statistically significant (*p* = 0.34).

**Fig. 6:**
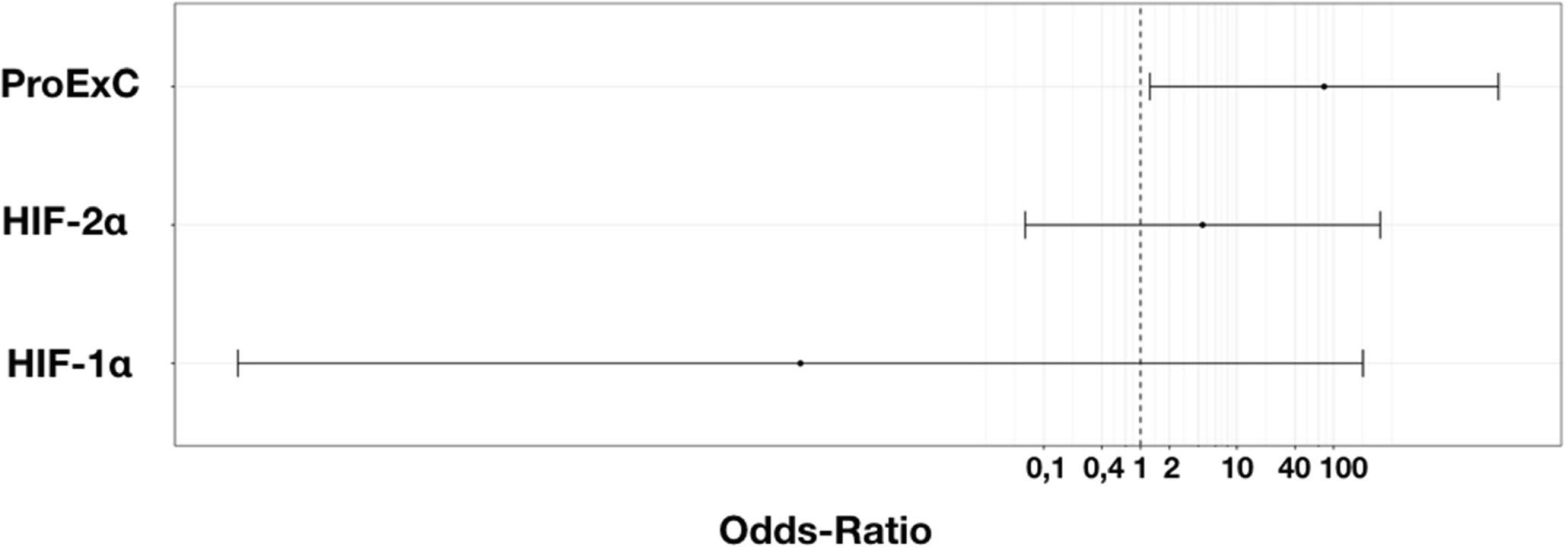
Odds ratio for ProExC, HIF-2α, and HIF-1α as influencing factors on tumor-related events. *X*-axis: odds ratio (OR), dots: Hazard ratio, horizontal line: 95% confidence interval

However, the Cox proportional hazards showed a significant elevated hazard ratio for staining with ProExC compared with HIF (Table 3 and Fig. 6).

**Table 3:** Cox proportional hazards for characterization of significant factors influencing the combined endpoint

Parameters	Hazard ratio	*p* value
HIF-1α	0.003	0.26
HIF-2α	4.41	0.49
ProExC	79.91	0.04

## Discussion

HIF-1α and HIF-2α are known to drive gene expression that support metabolism under hypoxic conditions. The activation of HIF-1α and HIF-2α seems to be common in malignancies [9]. Talks et al. described an overexpression of HIF-1α and HIF-2α in breast, colon, ovarian, pancreatic, prostate, renal, and hepatocellular carcinomas [8]. Moreover, No et al. have shown a notably increased expression of HIF-1α at higher cervical IN grades [5] and Birner et al. observed that HIF-1α was highly expressed in cervical cancer and in high-grade CIN compared with normal cervix [7]. Furthermore, clinical studies have revealed that higher expression of HIF-1α and HIF-2α seems to be associated with a poorer prognosis in many tumors [9]. In contrast, HIF is also able to reduce tumor growth by cell cycle arrest and control of proapoptotic genes [17, 18].

The present study showed low expression of HIF-1α and HIF-2α in CIN. The conjunctiva is well supplied with blood and has a high degree of external humidification and oxygen supply. In accordance to the cancer stem cell hypothesis, it is assumed that cells carrying the best situation-related mutation (“survival of the fittest”) accumulate in tumors [19]. We assume that HIF is downregulated to avoid cell cycle arrest and HIF-induced apoptosis because all cells of the conjunctiva may be well oxygenated, even if dysplastic. Schoelles et al., different investigators from our eye center, also analyzed HIF-1α and HIF-2α and the controls were treated in the same way as the specimens of our study. Their unpublished results include a higher expression of HIF-1α and HIF-2α in the controls compared with pterygium specimens. In contrast, we observed that the expressions of HIF-1α and also of TKTL-1 (transketolase-like protein 1), induced by hypoxia and HIF-1α, were higher in SCC of the ocular adnexa (including the conjunctiva) in comparison with conjunctival papilloma. Higher HIF-1α and TKTL-1 expression tended to be associated with a worse prognosis [16, 20, 21]. In the current study, we only stained the initial CIN specimens but not the later CIN recurrences or SCC. In order to investigate these contradictory findings further, a longitudinal analysis of HIF or TKTL-1 expression is warranted.

The significantly increased expression of HIF in normal samples compared with CIN was unexpected, given the external oxygen supply. Our analysis hint towards intraoperative hypoxia in the controls 20–45 min by many severed capillaries before the control specimen was completely separated from the adjacent conjunctiva at the end of the surgery. The expression of HIF-1α level of brain tissue increased significantly under experimental conditions in rats within less than 1 h after traumatic head injury [22]. For this reason, we speculate that the higher expression of HIF in the control specimens may be caused by a longer preexcisional hypoxia compared with the included CIN cases, which in contrast to the control specimens were immediately excised completely.

Furthermore, we investigated ProExC as a potential indicator for the classification of conjunctival specimens such as CIN and the course of the disease, but not as a reliable diagnostic marker. ProExC, a mix of two monoclonal antibodies against TOP2A and MCM2, has already been tested as a sufficient marker for proliferating cells and aberrant S-phase.

In our study, no significant difference in the proportion of ProExC-positive cells in CIN compared with normal conjunctival specimen was found (*p* = 0.55). This is in contrast to the findings of Guo et al. who investigated cervical specimens and showed a staining in over 50% of the dysplastic cells in high-grade cervical IN [6]. Nevertheless, our study revealed a tendency towards a more pronounced staining in specimens with recurrence or progression to SCC in the clinical course, but without statistical significance. This is in line with the results found in cervical IN and cervical carcinomas [6]. Regarding significance, it must be considered that a sample size consisting of three cases with progression to SCC is very small. Interestingly, all 3 patients progressing into SCC had autoimmune dermatitis (case 5 and 6 had atopic dermatitis and case 4 psoriasis) indicating smaller risk of patients without such an immune deviation to progress from CIN to SCC. However, compared with HIF-1α and HIF-2α, there was a statistically significant higher hazard ratio for ProExC staining within the first 4 years for CIN to develop a recurrence or SCC (*p* = 0.04). Evaluation of the vertical extension of ProExC staining showed that upper epithelial cells were more frequently stained in CIN compared with normal conjunctiva or metaplasia, however missing statistical significance (*p* = 0.06 and *p* = 0.07). Furthermore, we observed a statistically significant correlation between the proportion of ProExC-positive tumor cells and the vertical extension (*p* < 0.001).

A major drawback of our study is the low sample size. Our mostly negative findings may be due to a statistical power issue. Other limitations are the retrospective nature and the monocentric setting. Therefore, any clinic-related bias cannot be ruled out. However, a prospective large multicentric study is hard to perform because the conditions are very rare.

In conclusion, neither HIF-1α nor HIF-2α seems to be suitable as a diagnostic or prognostic marker in CIN. ProExC may be of some value for predicting recurrences or progression to SCC when analyzing the proportional staining. These findings should be confirmed in patients from other eye centers with a larger sample size.
